# Resistance Patterns of Frequently Applied Antimicrobials and Occurrence of Antibiotic Resistance Genes in *Edwardsiella tarda* Detected in Edwardsiellosis-Infected Tilapia Species of Fish Farms of Punjab in Pakistan

**DOI:** 10.4014/jmb.2301.01008

**Published:** 2023-02-17

**Authors:** Kashif Manzoor, Fayyaz Rasool, Noor Khan, Khalid Mahmood Anjum, Shakeela Parveen

**Affiliations:** 1 Department of Fisheries and Aquaculture, University of Veterinary and Animal Sciences, Lahore 54600, Pakistan; 2 Department of Zoology, Faisalabad Campus, University of Education, Lahore 54590, Pakistan; 3 Institute of Zoology, University of the Punjab, Lahore 54590, Pakistan; 4 Department of Wildlife and Ecology, University of Veterinary and Animal Sciences, Lahore 54600, Pakistan; 5 Department of Zoology, Wildlife and Fisheries, University of Agriculture, Faisalabad 38000, Punjab, Pakistan

**Keywords:** *Oreochromis niloticus*, antimicrobial susceptibility, Kirby-Bauer disk diffusion method, Mueller-Hinton agar (MHA), abnormalities, exophthalmia

## Abstract

*Edwardsiella tarda* is one of the most significant fish pathogens, causes edwardsiellosis in a variety of freshwater fish species, and its antibiotic resistance against multiple drugs has made it a health risk worldwide. In this study, we aimed to investigate the antibiotic resistance (ABR) genes of *E. tarda* and establish its antibiotic susceptibility. Thus, 540 fish (299 *Oreochromis niloticus*, 138 *O.mossambicus*, and 103 *O. aureus*) were collected randomly from twelve fish farms in three districts of Punjab in Pakistan. *E. tarda* was recovered from 147 fish showing symptoms of exophthalmia, hemorrhages, skin depigmentation, ascites, and bacteria-filled nodules in enlarged liver and kidney. Antimicrobial susceptibility testing proved chloramphenicol, ciprofloxacin, and streptomycin effective, but amoxicillin, erythromycin, and flumequine ineffective in controlling edwardsiellosis. Maximum occurrence of qnrA, blaTEM, and sul3 genes of *E. tarda* was detected in 45% in the liver, 58%, and 42% respectively in the intestine; 46.5%, 67.2%, and 55.9% respectively in *O. niloticus*; 24%, 36%, and 23% respectively in summer with respect to fish organs, species, and season, respectively. Motility, H_2_S, indole, methyl red, and glucose tests gave positive results. Overall, *E. tarda* infected 27.2% of fish, which ultimately caused 7.69% mortality. The Chi-squared test of independence showed a significant difference in the occurrence of ABR genes of *E. tarda* with respect to sampling sites. In conclusion, the misuse of antibacterial agents has led to the emergence of ABR genes in *E. tarda*, which in association with high temperatures cause multiple abnormalities in infected fish and ultimately resulting in massive mortality.

## Introduction

The rapid expansion of the global aquaculture industry has provided high-quality food products, more job opportunities, and economic benefits [1]. Meanwhile, aquatic organisms are an excellent source of life-sustaining proteins and nutrients [2, 3]. Fish and its various related products help to overcome food insecurity amidst a rapidly increasing human population. For this reason, fish has also become a crucial part of a sustainable diet from a future perspective [4]. Recent fish consumption worldwide is 20.5 kg per capita [5]. Tilapia is considered an economically and commercially important fish due to its high consumption rate, fast growth performance, efficient feed conversion ratio (FCR), high resistance against disease, and easy breeding nature [6]. These factors have positioned tilapia as the cheapest source of animal protein worldwide [7]. However, the culture of tilapia has also contributed to the emergence of antibiotic-resistant (ABR) bacteria [8]. At present, fish diseases caused by pathogenic bacteria pose a significant threat to aquaculture economics [9].

The close interaction between naturally resistant bacteria of terrestrial and aquatic environments contributes to the rapid transfer of ABR genes to pathogenic bacteria in fish [10]. So, fish is considered a vehicle for the dissemination of ABR bacteria and ABR genes [11]. Fish farmers employ multiple antibiotics to overcome fish mortality caused by ABR bacteria [12]. Their repeated and useless application increases antimicrobial-resistant (AMR) bacteria and their AMR genes in aquaculture [13]. ABR bacteria are an emerging and significant challenge to public health [14] as they are able to utilize mechanisms of genetic strategy by which they become resistant to the effects of antibiotics [15].

Edwardsiellosis is an enteric systemic disease in fish characterized by hernia, ascites, exophthalmia, hemorrhages, and severe lesions of the internal organs [16]. Edwardsiellosis outbreaks have caused significant losses at farms of various fish species in aquaculture [17]. Its causative agent, *Edwardsiella tarda*, is a gram-negative bacterium, a facultatively intracellular pathogen, and one of the emerging top causative pathogens [18, 19] of systemic hemorrhagic septicemic infection in fish, namely edwardsiellosis and hemorrhagic septicemia [16, 20, 21]. *E. tarda* has caused mass mortality in a wide variety of wild and cultured fish species of both marine and freshwater environments worldwide [22], especially in *Oreochromis niloticus*, *Cyprinus carpio*, *Labeo rohita*, *Clarias gariepinus*, *Seriola quinqueradiata*, *Anguilla japonica*, and *Pagrus major* [20, 23, 24]. The huge economic loss caused by *E. tarda* in commercially important fish species is due to its high resistance against multiple antibiotics [25, 26] as well as transmission of resistance from antibiotic-resistant *E. tarda* strains to non-resistant *E. tarda* strains [24, 27]. These ABR strains have antimicrobial resistance and virulence genes which cause pathogenicity [25, 21] and severe outbreaks, especially at tilapia and catfish farms [19, 28]. *E. tarda* is an opportunistic pathogen and infects fish under environmental stress, low water quality, high temperature and organic content, and stocking density [26, 29]. *E. tarda* poses high zoonotic risks [17] and infects a broad range of host animal species [20], including birds, reptiles, amphibians, mammals, and even humans [30-32].

In the current study, using conventional PCR, we sought to determine antibiotic susceptibility and check the occurrence of ABR genes in *E. tarda* detected in tilapia in the fish farms of Punjab, Pakistan.

## Materials and Methods

### Sample Collection, Clinical, and Postmortem Examination

**Fig. 1:**
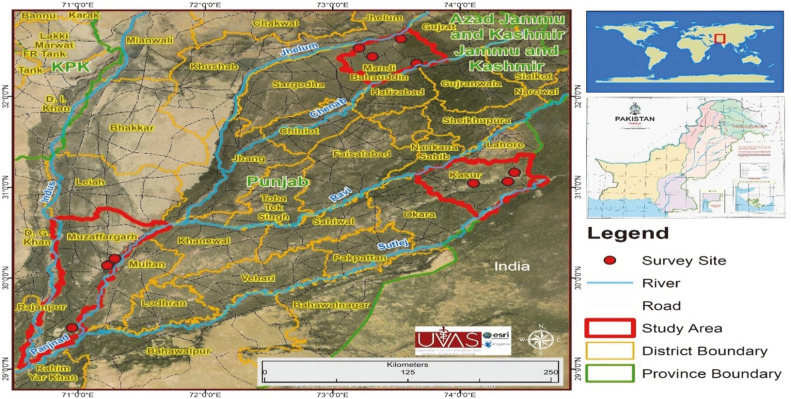
GIS map shows sampling sites (fish farms) of three selected districts of Punjab.

We randomly collected a total of 540 samples of tilapia fish species, viz., *Oreochromis niloticus*, *O. aureus*, and *O. Mossambicus* from selected fish farms of the districts of Kasur, Muzaffargarh, and Mandi-Bahauddin of Punjab in Pakistan. A GIS map showing the sampling sites (fish farms) in three districts is shown in Fig. 1. Ice-treated fish samples were transported to the laboratory of the Department of Fisheries and Aquaculture, University of Veterinary and Animal Sciences, Lahore, Pakistan. Suspected fish samples were examined for external and internal abnormalities during clinical and postmortem examination [33].

### Isolation, Phenotypic Characterization and Biochemical Identification of *E. tarda*

Suspected fish samples were disinfected with 70% ethanol and swabs from isolated organs (kidney, gills, liver, spleen, stomach, intestine, heart, and tail fins) were collected and inoculated onto trypticase soy agar (TSA, Oxoid, England) media plates and incubated at 37°C overnight. A single colony from freshly prepared culture was inoculated onto brain heart infusion agar (BHIA LAB, UK) media plates to obtain a pure culture of *E. tarda*, which was then incubated at 37°C for 24 h [34]. The pure culture of *E. tarda* isolates was stored at -20°C. A single colony from a freshly prepared *E. tarda* culture was subjected to phenotypic characterization and biochemical identification [35].

### Isolation of DNA

DNA was extracted using a Genomic DNA Purification Kit (Thermo Scientific, GeneJET, USA) and quantified spectrophotometrically (Nanodrop, USA). DNA samples were evaluated by gel electrophoresis on 1% agarose gel stained with ethidium bromide (C_21_H_20_BrN_3_) and utilizing a standard-sized molecular marker [1Kb DNA Ladder RTU (Ready-to-Use) GeneDireX, Taiwan]. Isolated DNA was stored at -20°C for further use.

### Molecular Detection of ABR Genes in *E. tarda* by PCR

*E. tarda* was detected by amplification of ABR genes (qnrA, blaTEM, and sul3) and gyrB gene by PCR (Bio-Rad, USA) using *E. tarda*-specific primers (Macrogen, Korea). A total of 25 μl of PCR reaction solution containing 1 μl of template DNA, 1 μl forward primer, 1 μl reverse primer, 10 μl injection water, and 12 μl GoTaq Green Master Mix (Promega, USA) were used to detect the ABR genes of *E. tarda* under amplification conditions for PCR as mentioned in Table 1. Amplified PCR products were analyzed on 1% agarose gel stained with ethidium bromide (C_21_H_20_BrN_3_) and utilizing a standard-sized molecular marker (1Kb DNA Ladder RTU, GeneDireX). PCR products revealing the thickest bands were sequenced by Sanger’s method at BGI Hong Kong Co. Ltd., China.

### Amplification, Sequencing, and Phylogenetic Tree Analysis of 16SrRNA Gene of *E. tarda*

One microliter of template DNA was added into a total of 25 μl reaction solution for PCR containing two primers of 16S rRNA gene; 1 μl forward primer (27F): AGAGTTTGATCCTGGCTCAG, 1 μl reverse primer (1492R): GGTTACCTTGTTACGACTT, 10 μl injection water and 12 μl GoTaq Green Master Mix (Promega, USA), under amplification conditions for PCR as mentioned in Table 1. PCR products were electrophoresed in 1%agarose gel stained with ethidium bromide (C_21_H_20_BrN_3_) and utilizing a standard-sized molecular marker (1Kb DNA Ladder RTU, GeneDireX). PCR products revealing the thickest bands were sequenced by Sanger’s method at BGI Hong Kong Co. Ltd., China. The obtained sequences were analyzed and compared for taxonomic identification using NCBI BLAST and submitted to the GenBank database. The phylogenetic relationship of *E. tarda* was checked by phylogenetic tree analysis of 16SrRNA gene of *E. tarda* by the bootstrap method using MEGA 11.0 (Molecular Evolutionary Genetic Analysis) with 1,000 bootstrap replications [36].

### Antimicrobial Susceptibility Testing

The Kirby-Bauer disk diffusion method was applied to determine the antibiotic susceptibility pattern using Mueller-Hinton agar (MHA) plates [37]. A total of 14 antibiotic discs were applied on MHA plates with a suspension of *E. tarda* colonies containing 1.5 × 10^8^ CFU/ml. Thereafter, 6 mm discs with discrete doses (5–30 μg) of antibiotics (Oxoid, UK), as mentioned in Table 8, were applied onto the MHA plate surfaces and incubated at 37°C for 24 h. The inhibition zones were measured to classify bacteria as resistant, moderately susceptible, and susceptible [38].

### Histopathological Characterization of Edwardsiellosis

Tissues specimens were collected from liver, stomach, and small intestine of infected fish. These collected tissue specimens were examined for histopathological changes due to edwardsiellosis and were treated by 10% neutral buffered formalin solution for one day to fix them. The fixed tissue specimens were submitted to the laboratory of the Department of Pathology, University of Veterinary and Animal Sciences, Lahore, Pakistan for histopathological examination.

### Statistical Analysis

Descriptive statistics such as proportions and percentage (%) were applied to summarize the data of prevalence. Chi-squared test of independence was applied to compare the occurrence of ABR genes of *E. tarda* with respect to fish species, organs of isolation, seasons, sampling sites, and fish sex using Statistical Package for Social Sciences (SPSS) version 21.0 software (IBM, USA).

## Results

### Isolation and Characterization of *E. tarda*

Swabs were collected from the organs of 540 fish samples and *E. tarda* was isolated by direct plating on TSA plates and BHIA plates. Quinolone resistance gene (qnrA), beta-lactam resistance gene (blaTEM), and sulphonamide resistance gene (sul3) of *E. tarda* were detected in 540 samples of three tilapia species (*O. niloticus*, *O mossambicus* and *O. aureus*) sampled from April to December. Colonies from a pure culture of *E. tarda* isolates showed phenotypic characteristics as opaque, large, irregular, translucent, and whitish-colored colonies on BHIA media plates while showing circular and round with grayish white-colored colonies on TSA media plates. Microscopic examination of *E. tarda* colonies revealed *E. tarda* as a motile and rod-shaped bacterium. Production of O_2_ caused bubble formation in the catalase test; red color appeared in Gram-staining, methyl red, and indole production test; diffused and hazy growths spread throughout the medium in the motility test, while black and purple-black color appeared in the H_2_S production and amylase tests, respectively. In addition, yellow color developed in the glucose fermentation test while no color change appeared in the arginine dihydrolase, cytochrome oxidase, citrate utilization, lactose fermentation, and Voges-Proskauer tests.

### Occurrence of ABR Genes in *E. tarda*

ABR genes of *E. tarda* (qnrA, blaTEM, and sul3) were amplified using *E. tarda*-specific primers and their amplification by PCR revealed bands at 801, 654, and 444, respectively. Maximum occurrence of qnrA was recorded in 45% of liver, while that of blaTEM and sul3 was found in 58% and 42% respectively in intestine, with respect to fish organs. Similarly, maximum occurrence of qnrA, blaTEM and sul3 was recorded in 46.5%, 67.2%, and 55.9% respectively in *O. niloticus* with respect to fish species, while 24%, 36%, and 23% were recorded respectively in summer with respect to season. Meanwhile, 32.3%, 56.2%, and 18.7% were recorded respectively in female fish samples with respect to fish sex, and 36%, 52%, and 26% was recorded respectively at 37°C and pH 6.67 (Tables 3-7). Chi-squared test of independence showed a significant difference (*p* < 0.05) in the occurrence of ABR genes of *E. tarda*, with respect to sampling sites, while insignificant (*p* > 0.05) differences were shown with respect to fish species, sex, and organs of isolation and season (Table 8). Accession numbers allotted by the NCBI GenBank database against all detected genes are given in Table 9.

### Overall Prevalence of *E. tarda* and Fish Mortality

**Fig. 2:**
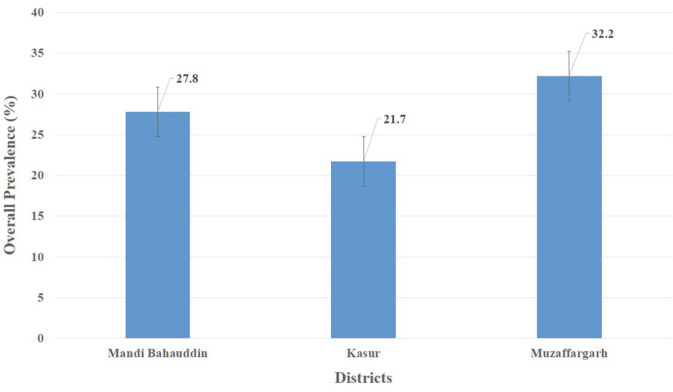
Overall prevalence (%) of *E. tarda* at selected districts.

**Fig. 3:**
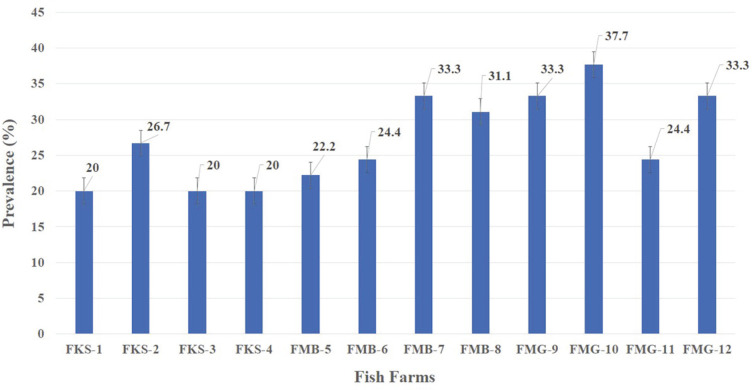
Overall prevalence (%) of *E. tarda* at selected fish farms of three districts (FKS: fish farms of district Kasur, FMB: Fish farms of Mandi Bahauddin, FMG: Fish farms of Muzaffargarh).

**Fig. 4:**
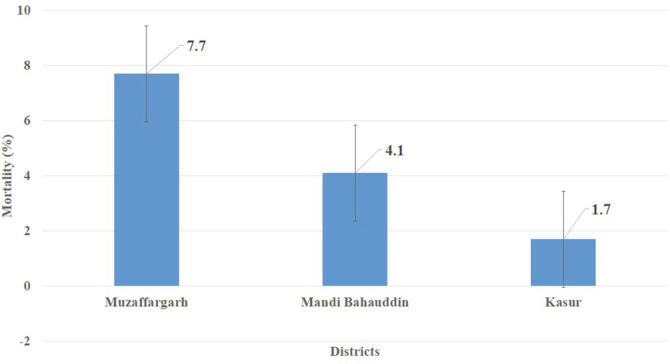
Mortality (%) in tilapia fish of all selected fish farms of three districts. Fig.

Overall, *E. tarda* infected 27.2% of fish (Fig. 2), but a maximum prevalence of 37.8% was recorded at a fish farm in Muzaffargarh Province designated as FMG-10, and minimum prevalence of 20% was seen at fish farms of Kasur Province, designated as FKS-1, FKS-3, and FKS-4 (Fig. 3). *E. tarda* caused 7.69% overall mortality (Fig. 4).

### Antimicrobial Susceptibility Test

This test was performed on 147 isolates of *E. tarda*. Chloramphenicol, ciprofloxacin, gentamicin, norfloxacin, streptomycin, and sulfamethoxazole were found 100% effective in controlling edwardsiellosis, but amoxicillin, erythromycin, flumequine, and neomycin were found ineffective to control *E. tarda* infection, and all *E. tarda* isolates showed 100% resistance. Whereas, all *E. tarda* isolates showed intermediate resistance (50%) against ampicillin, cefotaxime, tetracycline, and doxycycline (Table 8).

### Clinical and Post-Mortem Examination

**Fig. 5:**
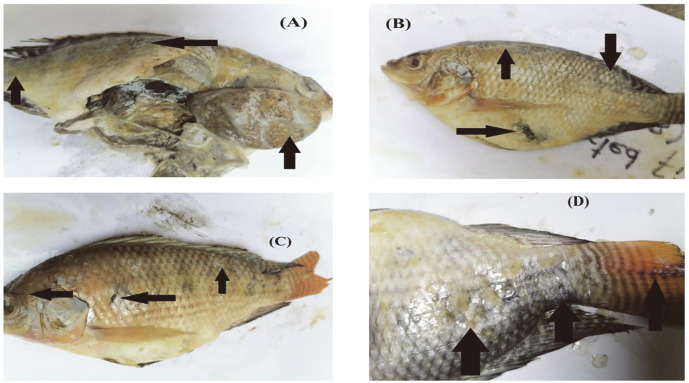
Clinical and post-mortem abnormalities. (**A**) enlarged liver with black dots and white bacteria filled nodules, (**B**) skin depigmentation, exophthalmia, and swollen abdomen, (**C**) hemorrhages, (**D**) scale loss and lesions on head, vent and tail.

Clinical and post-mortem examination revealed *E. tarda* infection, or edwardsiellosis, in 147 fish samples (27.2% prevalence). Infected fish showed a variety of external and internal abnormalities, such as hemorrhages, exophthalmia, dark spots, and skin lesions. Congested gills, vent, and fins, hemorrhaged and protruded anus, hernia, and swollen abdomen filled with ascitic fluid (ascites) were also observed in infected tilapia. White, bacteria-filled nodules were observed in the enlarged liver, intestine, gills, spleen and kidney (Fig. 5).

### Phylogenetic Tree Analysis

**Fig. 6:**
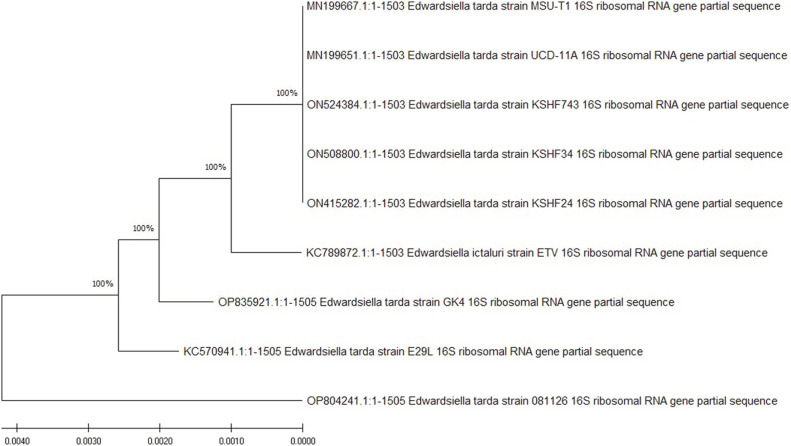
Phylogenetic tree of 16S rRNA gene of *E. tarda* by using the Neighbour-joining bootstrap method with 1000 bootstrap replicates.

The phylogenetic tree revealed that our isolated *E. tarda* strains, such as KSHF743 (ON524384), KSHF34 (ON508800), and KSHF24 (ON415282) shared 100% similarity and also 100% with previously isolated *E. tarda* strain UCD-11A (MN199651, USA), MSU-T1 (MN199667, USA), GK4 (OP835921, India), E29L (KC570941, China), and 081126 (OP804241, China). Phylogenetic tree analysis of the 16S rRNA gene of *E. tarda* is shown in Fig. 6.

### Histopathological Characterization of Edwardsiellosis

**Fig. 7:**
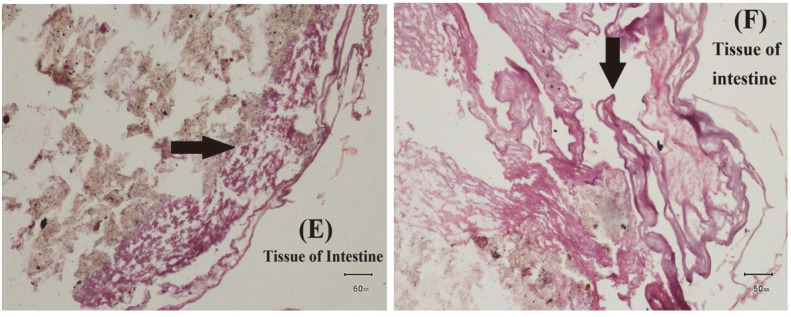
Examination of Histopathological Impact of *E. tarda*. (E) Mild cellular swelling in tissue of intestine (F) Artefactual changes in tissue of intestine.

**Fig. 8:**
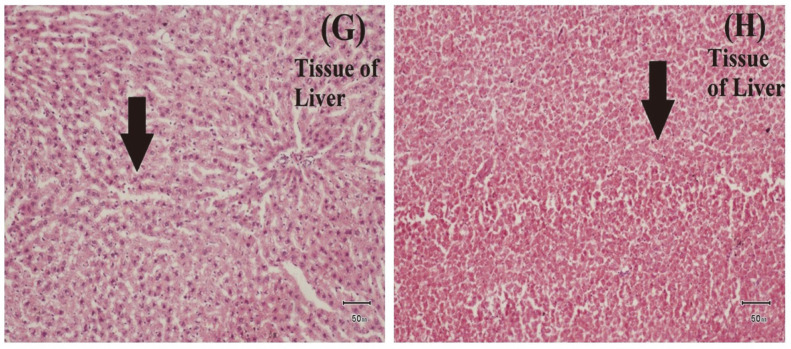
Examination of Histopathological Impact of *E. tarda*. (G) and (H) Cellular swelling in many hepatocytes of tissue of liver.

**Fig. 9:**
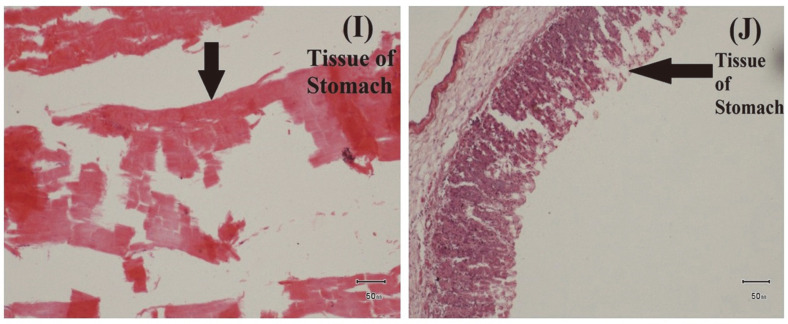
Examination of Histopathological Impact of *E. tarda*. (I) Sloughing and necrosis of gastric epithelial cells, (J) Some mononuclear inflammatory cells in tissue of stomach.

**Fig. 10:**
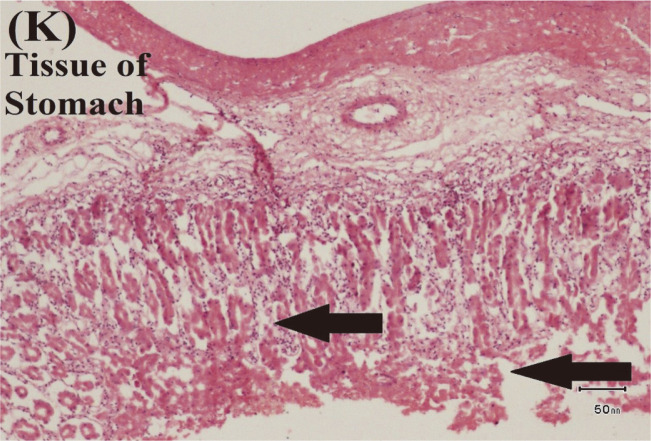
Examination of Histopathological Impact of *E. tarda*. (K) Artefactual changes in tissue of stomach.

*E. tarda* causes severe changes in tissues of edwardsiellosis-infected fish samples. Sloughing and necrosis of gastric epithelial cells were observed in the stomach tissues of infected fish. Some mononuclear inflammatory cells and artefactual changes were also observed in tissues of the infected stomach (Figs. 9 and 10). In addition, artifactual changes and cellular swelling were observed in the infected intestine (Fig. 7). Mild cellular swelling was seen in many hepatocytes of the infected liver (Fig. 8).

## Discussion

Edwardsiellosis has been reported in a wide variety of fish species worldwide and caused massive mortality in farmed and wild fish under stress [43]. Various antibiotics are being applied as one of the basic measures to control bacterial infections in aquaculture [44]. However, useless and uncontrolled application of antibiotics has enabled harmful bacteria to develop ABR genes while also giving rise to multiple antibiotic-resistant strains of pathogenic bacteria having ABR genes [45]. Such bacteria have caused severe outbreaks and high mortality at fish farms, ultimately resulting in huge economic losses for fish farmers [42]. Hence, routine antimicrobial susceptibility testing is vital to select suitable antibiotics to control the problem [45].

The acquisition of resistance against multiple antibiotics and the emergence of ABR genes in *E. tarda* can result in genetic mutations that may alter the target site of antibiotics or provide alternative pathways for survival against antibiotics with efficacy against gram-positive bacteria. Regarding the antibiotic susceptibility testing, retrieved *E. tarda* isolates revealed a significant difference in their sensitivity to different applied antibiotics. In the present study, chloramphenicol, ciprofloxacin, gentamicin, norfloxacin, streptomycin, and sulfamethoxazole were found 100% effective in controlling edwardsiellosis. However, in prior studies, *E. tarda* was observed sensitive to chloramphenicol, gentamicin, norfloxacin, streptomycin [26, 46], and ciprofloxacin [47]. Similarly, in the current study, amoxicillin, erythromycin, and flumequine were found 100% ineffective to control *E. tarda* while all *E. tarda* isolates showed intermediate resistance (50%) against tetracycline, ampicillin, cefotaxime, and doxycycline. Whereas, a prior study found tetracycline ineffective to control edwardsiellosis [46]. However, previous studies observed *E. tarda* isolates resistant to ampicillin [48, 49]. *E. tarda* isolates were resistant to tetracycline, erythromycin, and streptomycin [50], which may be due less to the application of these antibiotics than the increase in fish farming, high stocking density, application of animal waste as fertilizer, naturally antibiotic-resistant aquatic bacteria, and also the transfer of ABR genes from livestock and humans to fish [10, 51]. Recently, in another study, all *E. tarda* isolates were found susceptible against tetracycline, chloramphenicol and ciprofloxacin, and resistant to erythromycin, ampicillin, cefotaxime, and sulfamethoxazole, with intermediate resistance results against streptomycin, and gentamycin [28]. *E. tarda* had qnrA and sul3 genes but still *E. tarda* was found sensitive against ciprofloxacin and sulfamethoxazole, which may be due to the presence of other ABR genes, multiple resistance mechanisms, mutations, and high exposure to these antibiotics. Previously, *E. tarda* isolates were found resistant to amoxicillin and norfloxacin [26, 47, 52]. Similarly, neomycin was found effective in controlling edwardsiellosis [24, 52], but in our study, all *E. tarda* isolates showed intermediate resistance against neomycin. These differences in susceptibility might be due to different frequencies and quantities of applied antibiotics and also to varying *E. tarda* strains.

The existence of ABR genes, whether plasmid or chromosome, can be demonstrated using plasmid curing studies [21]. Recently, in a previous study, beta-lactam resistance gene blaTEM was detected in *E. tarda* and amplified at 60°C and sul3 at 53°C [27], while blaTEM gene was amplified at 52°C and sul3 gene at 54°C in the current study. This difference of 8°C and 1°C respectively might be due to variations in *E. tarda* strains with different antibiotic resistance profile, variation in application of beta-lactams and sulphonamides, and also different geographical distribution. Our recorded occurrence of β-lactam resistance gene (blaTEM), was nearly in accordance with findings of [53]. High occurrence may be due to the reason of their chromosomal-mediated, intrinsic resistance and fast transmission into the next pathogenic bacterial generations [54]. In the current study, 16S rRNA was amplified at 1,503 bp at 52°C for 1 min while previously, 16S rRNA bands were detected at 56°C for 30 s [25], at 1,465 bp size [55]. Currently, our isolated *E. tarda* strains revealed 100% similarity with *E. tarda* strains isolated in China, India, and the USA. Recently, in a previous study, similarity of 16S rRNA gene was recorded at 75%, 55% and 48% similarity [21], while 97% was reported by [27]. In the current study, *E. tarda* isolates showed positive results in motility, methyl red, H_2_S and indole production tests, while negative results were shown in gram-staining, citrate, lactose, amylase and urease tests. Similar results of biochemical identification of *E. tarda* were reported in previous studies [57, 58]. With respect to phenotypic characterization, the retrieved *E. tarda* isolates showed specific biochemical profiles, culture conditions, and phenotypic characteristics of *E. tarda* [59, 60]. Both biochemical and phenotypic characteristics are key factors for differentiation between *E. tarda* and other members of Enterobacteriaceae [61]. However, various studies showed variations in biochemical and phenotypic characteristics of *E. tarda* isolates [62]. These variations were attributed to the presence of plasmid that controls the metabolism in bacteria or to their ability to get energy for growth utilizing only citrate [63].

Severe outbreaks and massive mortality due to edwardsiellosis have been reported in farmed and wild fish under stress worldwide [43]. Our study concluded overall 7.69% mortality with 5.35% in *O. niloticus*, 4.52% in O. mossambicus and 3.47% in *O. aureus*. In prior studies, 40% mortality was recorded in *O. niloticus* of fish farms of Dakahlia Governorate, Egypt [19] while 60% was reported in infected fish of farms in Ernakulam, Kerala [21]. Similarly, 40% mortality was reported in autumn, 63.6% in winter, and 69.9% in spring [34]. This difference in fish mortality was due to variations in temperature, stocking density, water quality parameters and different geographical locality.

*E. tarda* has been associated with severe fish diseases, viz., hemorrhagic septicemia and lesions of skin and internal organs, thereby leading to massive mortality in various fish species [56]. In the current study, the results of clinical and post-mortem examination were in agreement with [20]. Moreover, the current post-mortem findings were highly compatible with those reported by [57], who observed muscular hemorrhage, ascitic fluid and congestion with small, white bacteria-filled nodules in enlarged spleen, kidney, and liver infected *O. niloticus*. Similarly, severe lesions were reported in the infected tilapia [64]. These variations in symptoms could be related to the pathogen’s ability to invade the fish immune status, severity of infection, site of sample collection, and other environmental conditions. Pathogenic bacteria cause infection in fish through oral and gill routes. In prior studies, histopathological changes were observed after experimental infection; however, the current study observed the impact of *E. tarda* infection in naturally infected fish tissues [26, 47, 65]. Moreover, in the current study many hepatocytes of infected liver showed cellular swelling while multifocal areas of necrosis were observed in infected liver [21]. Artifactual changes were also observed in intestine but lymphocytic enteritis was observed in intestine of infected fish [17].

The overuse of antibiotics in fish farms leads to the spread of antibiotic resistance and emergence of ABR genes, makes it more challenging to treat infections, especially edwardsiellosis in fish, which ultimately enhances the rate of pathogenicity of *E. tarda* and making it a major potential threat for tilapia culture. The antibiotic resistance pattern causes multiple abnormalities and severe infections in infected fish resulting in major outbreaks and ultimately causing massive mortality and huge economic loss for fish farmers. To mitigate this, antibiotics must be used responsibly and alternative strategies for disease control must be implemented.

## Figures and Tables

**Table 1:** Primer sequences and conditions for amplification of 16S rRNA and antibiotic resistance genes of *E. tarda* by PCR.

Target Gene	Primer sequence (5`-3`)	Amplified segment (bp)	Initial denaturation	Final denaturation	Annealing	Initial extension	Final extension	Reference
blaTEM	F-CATTTCCGTGTCGCCCTTATTC R-CGTTCATCCATAGTTGCCTGAC	800	95°C For 2 min	95°C For 30 s 35 cycles	52°C For 30 s 35 cycles	72°C For 1min 35 cycles	72°C For 7 min	[39]
qnrA	F-ATTTCTCACGCCAGGATTTG R-GATCGGCAAAGGTTAGGTCA	516	95°C For 10 min	95°C For 1min 35 cycles	58.5°C For 1min 35 cycles	72°C For 1min 35 cycles	72°C For 10 min	[40]
sul3	F-AGATGTGATTGATTTGGGAGC R-TAGTTGTTTCTGGATTAGAGCCT	443	93°C For 3 min	93°C For 30s 35 cycles	54.2°C For 30 s 35 cycles	72°C For 1min 35 cycles	72°C For 7min	[39]
16SrRNA gene	F-AGAGTTTGATCCTGGCTCAG R-GGTTACCTTGTTACGACTT	1503	94°C For 5 min	90°C For 30 s 30 cycles	52°C For 1min 30 cycles	72°C For 1min 30 cycles	72°C for 8 min	[41]
gyrB	F-GCATGGAGACCTTCAGCAAT R-GCGGAGATTTTGCTCTTCTT	414	94°C For 1 min	94°C For 1min 32 cycles	55°C For 1min 32 cycles	72°C For 1min 32 cycles	72°C For 10 min	[66]

**Table 2:** Occurrence of 16S rRNA and antibiotic resistance genes of *E. tarda* with respect to fish organs.

Organ	qnrA gene	blaTEM gene	sul3 gene	16SrRNA gene
Liver	135 (45%)	157 (52%)	122 (41%)	300 (100%)
Kidney	123 (41%)	143 (48%)	114 (38%)	284 (95%)
Spleen	118 (39%)	132 (44%)	98 (33%)	293 (98%)
Intestine	133 (44%)	174 (58%)	126 (42%)	297 (99%)
Stomach	67 (22%)	109 (36%)	42 (14%)	277 (92%)
Gills	41 (14%)	83 (28%)	37 (12%)	189 (63%)
Tail fins	18 (6%)	26 (9%)	12 (4%)	106 (35%)
Heart	26 (9%)	41 (14%)	20 (7%)	148 (49%)
Overall	83 (27.5%)	108 (36.04%)	71 (23.8%)	237 (78.9%)

**Table 3:** Occurrence of 16S rRNA and antibiotic resistance genes of *E. tarda* with respect to fish species.

Fish species	qnrA gene	blaTEM gene	sul3 gene	16SrRNA gene
*Oreochromis niloticus*	139 (46.5%)	201 (67.2%)	170 (56.9%)	183 (61.2%)
*O. Mossambicus*	51 (36.9%)	75 (54.3%)	63 (45.6%)	77 (55.8%)
*O. aureus*	46 (44.6%)	57 (55.3%)	34 (33.3%)	40 (38.8%)

**Table 4:** Occurrence of 16S rRNA and antibiotic resistance genes of *E. tarda* with respect to season.

Season	qnrA gene	blaTEM gene	sul3 gene	16SrRNA gene
Summer	36 (24%)	54 (36%)	34 (23%)	193 (64.3%)
Winter	4 (8%)	5 (10%)	3 (6%)	21 (7.0%)
Spring	10 (20%)	11 (22%)	9 (18%)	50 (16.7%)
Autumn	8 (16%)	8 (16%)	5 (10%)	36 (12.0%)

**Table 5:** Occurrence of 16S rRNA and antibiotic resistance genes of *E. tarda* with respect to fish sex.

Sex	qnrA gene	blaTEM gene	sul3 gene	16SrRNA gene
Male	52 (28.6%)	84 (46.1%)	32 (17.6%)	187 (62.3%)
Female	62 (32.3%)	108 (56.2%)	36 (18.7%)	113 (37.7%)

**Table 6:** Occurrence of 16S rRNA and antibiotic resistance genes of *E. tarda* with respect to water temperature (oC) and pH.

Month	Temperature (°C)	pH	qnrA gene	blaTEM gene	sul3 gene	16SrRNA gene
Apr-20	25.58°C	7.92	2 (8%)	3 (12%)	1 (4%)	11 (27.5%)
May-20	26.5°C	7.68	4 (16%)	6 (24%)	3 (12%)	23 (57.5%)
Jun-20	28.91°C	7.42	11 (22%)	14 (28%)	9 (18%)	48 (80.0%)
Jul-20	37°C	6.67	18 (36%)	26 (52%)	13 (26%)	73 (81.1%)
Aug-20	36.17°C	6.8	14 (28%)	18 (36%)	12 (24%)	67 (74.4%)
Sep-20	28.75°C	7.87	5 (20%)	6 (24%)	4 (16%)	36 (40.0%)
Oct-20	26.83°C	7.95	3 (12%)	4 (16%)	2 (8%)	20 (33.3%)
Nov-20	24.75°C	8.27	2 (8%)	3 (12%)	1 (4%)	14 (40.0%)
Dec-20	19.92°C	8.4	1 (4%)	2 (8%)	0 (0%)	8 (22.8%)

**Table 7:** Results of statistical analysis; chi-squared test of independence showing X^2^-value and p-value with respect to selected parameters.

Parameters	X^2^-value	*p*-value
Sampling sites	294	0.00***
Fish Farms	7.364	0.998^ns^
Season	0.233	1.00^ns^
Organs	3.262	0.999^ns^
Sex	0.844	0.656^ns^

*** = Shows significant result, ns = non-significant result

**Table 8:** Results of antimicrobial susceptibility of *E. tarda* isolates and MIC values.

Antibiotics (conc.)	Disc code	Concentration (μg)	Sensitive	Intermediate	Resistant	MIC_50_ (μg /ml)	MIC_90_ (μg /ml)
Amoxicillin	AMX	10	0	0	100%	>64	>128
Ampicillin	AMP	10	0	50%	50%	<16	<32
Cefotaxime	CTX	30	0	50%	50%	>8	>16
Chloramphenicol	C	30	100%	0	0	0.5	1
Ciprofloxacin	CIP	5	100%	0	0	2	4
Doxycycline	DO	5	0	50%	50%	8	16
Erythromycin	E	15	0	0	100%	>32	>64
Flumequine	FLU	30	0	0	100%	>64	>128
Gentamicin	GM	10	100%	0	0	0.5	1
Neomycin	N	30	0	50%	50%	>16	<32
Norfloxacin	NOR	10	100%	0	0	<2	<4
Streptomycin	S	10	100%	0	0	1	2
Sulfamethoxazole	SXT	25	100%	0	0	2	4
Tetracycline	TE	30	0	50%	50%	8	16

**Table 9:** Accession numbers of 16S rRNA and antibiotic resistance genes of *E. tarda*.

Gene	District	Accession number
*blaTEM*	Kasur	OP919343
	Mandi Bahauddin	OP919344
	Muzaffargarh	OP919345
*qnrA*	Kasur	OP901500
	Mandi Bahauddin	OP901501
	Muzaffargarh	OP901502
*sul3*	Kasur	OP919347
	Mandi Bahauddin	OP919348
	Muzaffargarh	OP919349
*16SrRNA*	Kasur	ON508800
	Mandi Bahauddin	ON415282
	Muzaffargarh	ON524384
*gyrB*	Kasur	OQ326581
	Mandi Bahauddin	OQ326582
	Muzaffargarh	OQ326583
